# Whole Genome Analysis of *Lactobacillus plantarum* Strains Isolated From Kimchi and Determination of Probiotic Properties to Treat Mucosal Infections by *Candida albicans* and *Gardnerella vaginalis*

**DOI:** 10.3389/fmicb.2019.00433

**Published:** 2019-03-06

**Authors:** Bo Ram Beck, Gun-Seok Park, Yong Hyun Lee, Sunghoon Im, Do Yeun Jeong, Jihee Kang

**Affiliations:** AtoGen Co., Ltd., Daejeon, South Korea

**Keywords:** *Lactobacillus plantarum*, probiotics, *Candida albicans*, candidiasis, *Gardnerella vaginalis*, bacterial vaginosis, macrophage, whole genome sequencing

## Abstract

Three *Lactobacillus plantarum* strains ATG-K2, ATG-K6, and ATG-K8 were isolated from Kimchi, a Korean traditional fermented food, and their probiotic potentials were examined. All three strains were free of antibiotic resistance, hemolysis, and biogenic amine production and therefore assumed to be safe, as supported by whole genome analyses. These strains demonstrated several basic probiotic functions including a wide range of antibacterial activity, bile salt hydrolase activity, hydrogen peroxide production, and heat resistance at 70°C for 60 s. Further studies of antimicrobial activities against *Candida albicans* and *Gardnerella vaginalis* revealed growth inhibitory effects from culture supernatants, coaggregation effects, and killing effects of the three probiotic strains, with better efficacy toward *C. albicans*. *In vitro* treatment of bacterial lysates of the probiotic strains to the RAW264.7 murine macrophage cell line resulted in innate immunity enhancement via IL-6 and TNF-α production without lipopolysaccharide (LPS) treatment and anti-inflammatory effects via significantly increased production of IL-10 when co-treated with LPS. However, the degree of probiotic effect was different for each strain as the highest TNF-α and the lowest IL-10 production by the RAW264.7 cell were observed in the K8 lysate treated group compared to the K2 and K6 lysate treated groups, which may be related to genomic differences such as chromosome size (K2: 3,034,884 bp, K6: 3,205,672 bp, K8: 3,221,272 bp), plasmid numbers (K2: 3, K6 and K8: 1), or total gene numbers (K2: 3,114, K6: 3,178, K8: 3,186). Although more correlative inspections to connect genomic information and biological functions are needed, genomic analyses of the three strains revealed distinct genomic compositions of each strain. Also, this finding suggests genome level analysis may be required to accurately identify microorganisms. Nevertheless, *L. plantarum* ATG-K2, ATG-K6, and ATG-K8 demonstrated their potential as probiotics for mucosal health improvement in both microbial and immunological contexts.

## Introduction

Mucosa or mucosal sites are external tissues of animals covered with mucus, which are exposed to lifespan-ensuring and/or transient microorganisms known as commensal microbiota. The gut is the most studied mucosal site, but other mucosa including air ways, nostril, oral cavity, and vagina also have been thoroughly studied with impacts in the microbiome and immunological fields (Gill et al., 2010). In general, mucosal sites are protected by mucus, secreted immunoglobulin A, antimicrobial peptides, and innate and adaptive immune cells from various non-self-antigens including commensal microbiota and food antigens (Tlaskalová-Hogenová et al., 2004; Brandtzaeg, 2009; McDermott and Huffnagle, 2014). Usually many microbes are tolerated under healthy conditions and are even associated with beneficial effects on immune development, organ development, metabolism, and homeostasis of the host (Sommer and Bäckhed, 2013).

However, some members of commensal microbiota such as *Escherichia coli, Staphylococcus* spp., *Streptococcus* spp. in mucosal sites can occasionally be pathogenic in certain cases depending on the type of microorganism and health status of the host, such as invasion of highly virulent microbes, dysbiosis of microbiota by antibiotics, mechanical damage of mucosa, and/or dysregulation of immune system of the host (Honda and Littman, 2012; Khosravi and Mazmanian, 2013). Among commensal microbe-associated mucosal infections related to dysbiosis, candidiasis is one of the more notorious infections, and occurs at most mucosal sites causing inflammatory symptoms and even systemic infection (candidemia) mainly in immunocompromised individuals (Kim and Sudbery, 2011; Moyes and Naglik, 2011). Candidiasis is closely related to the pathogenesis of vulvovaginal candidiasis (Sobel et al., 1998; Kim and Sudbery, 2011), Crohn’s disease (Poulain et al., 2009), oral infections (Ellepola and Samaranayake, 2000) and microbial infections in individuals with acquired immune deficiency syndrome (Kim and Sudbery, 2011). Another typical mucosal disease model caused by dysbiosis of microbiota is bacterial vaginosis (BV), an infectious disease with symptoms of bad odor, excessive discharge, erythema, increment of pH, and inflammation (Morris et al., 2001; Egan and Lipsky, 2002). BV is caused by (1) disruption of vaginal microbiota due to overdosed antibiotics treatment, stress, hormonal changes, and/or physical damage, (2) resultant loss of hydrogen peroxide producing lactobacilli and bifidobacteria, and (3) increased pH in the vagina that allows the growth of other opportunistic pathogens (Sobel, 1997).

To treat such microbial infections in mucosal sites, a probiotic approach is one of the important measures besides chemical or antibiotic treatments that may restore mucosal microbiota to a healthy distribution and/or eliminate certain pathogens in a microbiome environment. Mechanisms of probiotics toward microbial infections include bacteriocin, co-aggregation, production of hydrogen peroxide (H_2_O_2_), production of organic acids, reduction of pH, and immunomodulation, which may act singularly or synergistically to benefit the host (Reid et al., 2011).

With mechanistic criteria focused on treating and/or preventing mucosal infection as mentioned above, *Lactobacillus plantarum* ATG-K2, ATG-K6, and ATG-K8 were isolated in the present study from Kimchi, a Korean traditional fermented vegetable, and examined for their safety and functionalities in biologic and genomic analyses as potential probiotics for improving mucosal health.

## Materials and Methods

### Microorganisms and Their Culture Conditions

Three *Lactobacillus plantarum* strains, *L. plantarum* ATG-K2 (K2), *L. plantarum* ATG-K6 (K6), and *L. plantarum* ATG-K8 (K8) were isolated from kimchi samples, traditional Korean fermented vegetables, made in the Chungchung region of South Korea. *Lactobacillus* strains were cultured in de Man Rogosa Sharpe (MRS) medium (Difco Laboratories, United States) at 37°C. Each strain was isolated from different batches of kimchi samples. Lactobacilli were first identified as *L. plantarum* strains by 16S rRNA sequences using nucleotide BLAST tool of the National Center for Biotechnology Information (NCBI) then processed to whole genome sequencing of section Whole Genome Analysis of *L. plantarum* Strains of the present study.

For antibacterial effect testing of lactobacilli strains, *Staphylococcus aureus* KCTC1621 (SA), *Escherichia coli* KCTC1682 (EC), *Pseudomonas aeruginosa* KCTC2004 (PA), *Listeria monocytogenes* KCTC3569 (LM), *Cronobacter sakazakii* KCTC2949 (CS), *Streptococcus mutans* KCTC3065 (SM), *Streptococcus salivarius* ATG-P1 (SS) were used as representative opportunistic pathogens. Opportunistic pathogens were cultured in brain heart infusion medium (BHI, Difco Laboratories, United States) at 37°C.

To determine the impact of *L. plantarum* strains on infectious mucosal pathogens, *Candida albicans* KCTC7678 (CA) and *Gardnerella vaginalis* KCTC5096 (GV) were selected as representative candidiasis and BV pathogens, respectively. CA was cultured in yeast extract maltose medium (YM, Difco Laboratories, United States) at 37°C, and GV was cultured in BHI supplemented with 20% heat-inactivated horse serum (Gibco, United States) under anaerobic conditions using an Oxoid^TM^ AnaeroGen^TM^ system (Oxoid, United Kingdom) at 37°C.

### Characterization of *L. plantarum* Strains

#### Carbohydrate Fermentation Patterns of *L. plantarum* Strains

Fermentation patterns of *L. plantarum* strains were tested with an API 50 CHL test (BioMérieux, France) toward 49 selected carbohydrate sources following the manufacturer’s guidelines. Briefly, overnight cultures of lactobacilli were suspended in 10 mL API 50 CHL medium (BioMérieux, France) and each applied to cupels containing different carbohydrates on an API 50 CH test strip. Fermentation patterns were monitored for up to 72 h at 37°C.

#### Heat Resistance Test of *L. plantarum* Strains

To test heat resistance of *L. plantarum* strains, overnight broth-cultured strains were heated in a water bath at 70°C for 20, 40, 60, and 80 s. Viable bacteria from each time point were measured by 10-fold serial dilution and spread-plate method.

#### Antibacterial Effects of *L. plantarum* Strains

Antibacterial effects of *L. plantarum* strains were tested by the disc diffusion method. Four Gram-positive bacteria (SA, LM, SM, SS) and three Gram-negative bacteria (EC, PA, CS) were used as target bacteria. Overnight cultures of target bacteria were suspended in 0.9% saline with an optical density (OD) of 0.8 at 600 nm wavelength (OD_600_). Each bacterial suspension was spread on mixed agar medium of BHI and MRS at a 1:1 ratio in weight. After spreading bacterial suspensions, 8 mm paper discs (Advantec, Japan) were placed on agar plates, and 35 μL of each overnight lactobacillus broth culture was inoculated on the paper discs. After 24–48 h of incubation at 37°C, diameters of inhibition zones were measured. The diameter of the paper disc was subtracted from the total diameter of each measured inhibition zone.

#### Bile Salt Hydrolase (BSH) Activity of *L. plantarum* Strains

BSH activity of *L. plantarum* strains was determined using the method described by Dashkevicz and Feighner (1989). Briefly, each strain was streaked on MRS agar medium supplied with 0.5% w/v sodium taurocholic acid (TDCA, Sigma-Aldrich, Germany). Inoculated agar plates were placed in an anaerobic jar and incubated at 37°C for 48–72 h.

#### Hydrogen Peroxide (H_2_O_2_) Production of *L. plantarum* Strains

H_2_O_2_ production ability of *L. plantarum* strains was determined with 0.25 mg/mL tetramethylbenzidine (TMB, Sigma-Aldrich, Germany) and 0.01 mg/mL horseradish peroxidase (Sigma-Aldrich, Germany) in MRS agar medium as described by McGroarty et al. (1992). Additives were filtered through 0.2 μm size pore filter (Satorious, Germany) and added to autoclaved MRS agar medium when the medium reached approximately 50°C. Test specimens were incubated at 37°C for 48–72 h in an anaerobic jar, and exposed to air at 25°C to monitor color change of colonies.

#### Radical Scavenging Activity Test of Lysates of Each *L. plantarum* Strain

Radical scavenging activity of each *L. plantarum* strain was tested by using a 2, 2′-azino-bis 3-ethylbenzthiazoline-6-sulfonic acid (ABTS, Sigma-Aldrich, Germany) colorimetric assay. The test was conducted with lysates from each strain to avoid growth and to examine both intracellular and extracellular contents of the bacteria. To prepare bacterial lysates, biomass from overnight broth cultured strains were obtained by centrifugation at 4,000 × *g* for 30 min at 4°C. Bacterial pellets were washed twice with 1× phosphate buffered saline (PBS) and concentrated to 10× by resuspending in PBS. The samples were treated with lysozyme (Sigma-Aldrich, Germany) at 37°C for 2 h, then lysed with sonication. Solid content of resulting lysates was measured with a moisture analyzer (A&D Co., Ltd., Japan), and the final stock solution concentration was adjusted to 50 mg/mL. ABTS was prepared by mixing 14 mM ABTS and 4.9 mM potassium persulfate at a 1:1 ratio and incubating in a darkroom overnight. The OD of the solution was adjusted to 0.7 at 734 nm wavelength to prepare a working solution. Lysate samples were added into the working solution at a 1:10 ratio on microplates and incubated for 10 min in a darkroom. The ABTS-reacted samples were centrifuged at 13,000 rpm for 5 min at 4°C, and the resulting supernatants were measured at 734 nm wavelength with an Epoch microplate spectrophotometer (BioTek Instruments, United States). Percent ABTS radical scavenging activity was calculated using following equation, where OD*sample* is the measured OD value of ABTS-reacted lysate and OD*control* is the OD of non-reacted ABTS control:

$$\mathrm{ABTS radical scavenging activity}\;(\%)=\left\{1-\left(\frac{{\text{OD}}_{\text{sample}}}{{\text{OD}}_{\text{control}}}\right)\right\}\times 100$$

### Safety Assessment of *L. plantarum* Strains

#### Hemolysis Test

To determine the safety of *L. plantarum* strains in the present study, hemolysis activity was first examined with 5% sheep blood supplied in tryptic soybean agar (TSA, Difco Laboratories, United States) by streaking each strain onto the medium followed by overnight incubation at 37°C under anaerobic conditions. SA was also streaked onto a sheep blood agar medium as positive control for the experiment.

#### Antibiotics Susceptibility Examination

Antibiotic susceptibility of lactobacilli was tested with ampicillin, gentamicin, kanamycin, streptomycin, clindamycin, erythromycin, tetracycline, and chloramphenicol *E*-test strips (BioMérieux, France) to determine minimum inhibitory concentration (MIC) values. Briefly, overnight cultures of lactobacilli strains were suspended in 0.9% saline at an OD_600_ of 0.8. Bacterial suspensions were spread onto counting agar plates (PCA, Difco Laboratory, United States), and E-test strips were placed on the agar plates. MIC cut-off values were determined using European food safety authority (EFSA) guidelines on antibiotic susceptibility (EFSA, 2012).

#### Biogenic Amine Production Test

Production of the potentially harmful biogenic amines histamine, tyramine, putrescine, and cadaverine was examined using a test medium described by Bover-Cid and Holzapfel (1999). The test medium was made according to the composition of Bover-Cid and Holzapfel and each strain was streaked on the medium. The inocula were incubated at 37°C for 72 h, and colorimetric change of bromocresol purple in the medium from yellow to purple due to pH increase by decarboxylase activity (indicating a positive result) was monitored.

### Antimicrobial Effects of *L. plantarum* Strains on *C. albicans* (CA) and *G. vaginalis* (GV)

#### Growth Inhibition of Pathogens

Growth inhibition effects of cell free culture supernatant (CFCS) of *L. plantarum* strains toward CA and GV were measured. CFCS was prepared from overnight broth cultures of each strain by centrifugation at 5,000 rpm for 30 min. CFCS was filtered through 0.2 μm pore syringe filter for sterilization. CFCS was freeze dried and resuspended in 1× PBS to make a 10× stock solution. No additional process of CFCS was done after stock preparation to preserve all substances in the CFCS. CA was inoculated at 1 × 10^5^ CFU/mL in YM broth supplied with CFCS at 1× final concentration. GV was inoculated at 1 × 10^6^ CFU/mL in BHI broth supplemented with 20% heat inactivated horse serum and CFCS. Samples without CFCS were used as controls. CA was incubated at 37°C for 24 h and GV for 48 h depending on growth properties of each pathogen. Endpoint OD was measured at 600 nm wavelength by a spectrophotometer. Growth inhibition rates were calculated using following equation, where OD*control* and OD*cfcs* are the OD of the control and CFCS-treated groups, respectively.

$$\mathrm{Growth inhibition}(\%)=\left(\frac{{\text{OD}}_{\text{control}}-{\text{OD}}_{\text{cfcs}}}{{\text{OD}}_{\text{control}}}\right)\times 100$$

#### Coaggregation of *L. plantarum* Strains and Pathogens

Coaggregation activity to pathogens, another potential antimicrobial ability, was determined for the *L. plantarum* strains by a method described by Handley et al. (1987). Briefly, overnight broth-cultured microorganisms were collected by centrifugation at 13,000 rpm for 5 min at 4°C, and cell pellets were washed twice with 1× PBS. Each microorganism was resuspended in 1× PBS and adjusted to an OD value of 1.0 at 600 nm wavelength. *L. plantarum* strain and pathogen suspensions were mixed in equal volumes, vortexed for 5 min and left at room temperature for sedimentation. The upper part of the mixture was carefully removed and its OD was measured at 1, 4, and 8 h time points for each sample. Single suspensions of each microorganism were used as controls. Measured OD values were calculated with following equation to determine coaggregation rate, where OD*patho*, OD*lp*, and OD*mix* are the ODs of the pathogen groups, *L. plantarum* strains, and strain and pathogen mixture, respectively.

$$\text{Coaggregaion }(\%)=\left(\frac{{\text{OD}}_{\text{Patho}}+{\text{OD}}_{\text{lp}}}{\text{2}}-{\text{OD}}_{\text{mix}}\right)÷\left(\frac{{\text{OD}}_{\text{Patho}}-{\text{OD}}_{\text{lp}}}{\text{2}}\right)$$

#### Coculture of *L. plantarum* Strains and Pathogens

Coculture experiments of *L. plantarum* strains and pathogens were conducted to examine direct killing effects. Approximately 2.5 × 10^6^ CFU/mL of CA and of each strain of *L. plantarum* were inoculated in a 1:1 ratio of mixed YM and MRS broth and incubated at 37°C for 48 h. For the anti-GV test, approximately 1.0 × 10^8^ CFU/mL of GV and of each *L. plantarum* strain were inoculated in BHI broth supplemented with 20% horse serum, 1% yeast extract, and 0.1% maltose and incubated at 37°C for 48 h in an anaerobic jar. Each test inoculum was sampled and checked for viable pathogens by spread-plate method. For CA growth medium, 100 μg/mL ampicillin supplemented YM agar medium was used to grow CA selectively. For GV growth medium, 5% rabbit blood BHI agar medium was used and GV was selected based on white, slight translucent colony morphology and hemolysis zones formed around GV colonies.

### *In vitro* Immunomodulation of *L. plantarum* Strains

#### Innate Immunity Cytokine Induction in RAW264.7 Murine Macrophages

To determine whether *L. plantarum* strains are capable of immunomodulation, *in vitro* cell experiments with RAW264.7 murine macrophage cells were performed. Cells were cultured in Dulbecco Modified Eagle Medium (DMEM, Gibco, United States) supplemented with 10% fetal bovine serum (Gibco, United States) and 1% penicillin/streptomycin cocktail (Sigma-Aldrich, Germany) throughout the experiment. RAW264.7 cultured up to 80–90% confluency was collected and 1 × 10^6^ cells were seeded in each well of a 24 well cell culture plate (SPL Life Science, Korea). Seeded cells were stabilized at 37°C in 5% CO_2_ atmosphere for 24 h. Then, 1 μg/mL of lipopolysaccharide (LPS, Sigma-Aldrich, Germany) was administered was as administered to 100 or 500 μg/mL of lysates from each *L. plantarum* strain. Non-treated cells were used as a negative control and LPS-treated cell as a positive control. After treating with each substance, cells were incubated at 37°C in 5% CO_2_ atmosphere for 24 h. Resulting culture supernatants of each treatment group were collected and processed with IL-6 enzyme linked immunosorbent assay (ELISA) with a Mouse IL-6 Quantikine ELISA Kit (R&D Systems, United States) and TNF-α ELISA with a Mouse TNF-alpha Quantikine ELISA Kit (R&D Systems, United States) for quantification of produced cytokines.

#### Suppression of Inflammation Due to LPS Challenge by *L. plantarum* Strains

Using the same experimental procedure described previously in the present study with some modification, IL-10 production enhancement in macrophages by the lysate of *L. plantarum* strains was examined, and 1 μg/mL LPS and 100 μg/mL of *L. plantarum* lysate co-treated groups were added in this experiment. After treatment with each substance, cells were incubated for another 24 h, and culture supernatant samples were collected. IL-10 concentrations of each culture supernatant of the experimental groups were determined with an IL-10 Quantikine ELISA Kit (R&D Systems, United States). The dose of lysate (100 μg/mL) was selected as a representative dose based on the previous experiment of IL-6 and TNF-α quantification.

### Whole Genome Analysis of *L. plantarum* Strains

#### Genomic DNA Isolation and Whole Genome Sequencing

A single colony from each bacterial strain was inoculated in 3 mL MRS broth in a shaking incubator at 30°C for 6 h. Genomic DNA was extracted from the culture broths after 6 h using a Wizard Genomic DNA Purification Kit (Promega, Madison, WI, United States) according to the manufacturer’s instructions. Genomic DNA concentration was determined using a Qubit 2.0 Fluorometer (Thermo Fisher Scientific, Waltham, MA, United States) and the DNA quality and integrity were checked by electrophoresis on an 0.8% agarose gel. Whole genomes were sequenced on a Pacific Biosciences (Menlo Park, CA, United States) single molecule real-time (SMRT) sequencing platform with P6-C4 chemistry on a PacBio RS II instrument (Eid et al., 2009). The raw reads were generated with SMRT Cells and *de novo* assembled by the hierarchical genome-assembly process (HGAP) (Chin et al., 2013) protocol RS HGAP Assembly 2 in SMRT analysis version 2.3.0 (Pacific Biosciences) ^1^. *De novo* assembly parameters were applied as follows: PreAssembler v2: Minimum Seed Read Length: 5000, Celera Assembler v1 Genome Size (Bp): 3,500,000, Target Coverage: 30, Overlapper Error Rate: 0.06, Overlapper Min Length: 40, Overlapper K-mer: 14.

#### Gene Prediction and Genome Comparison

Complete genomes were annotated by NCBI Prokaryotic Genomes Automatic Annotation Pipeline (PGAAP) (Tatusova et al., 2016). PathogenFinder was used to determinate the pathogenic potential (Cosentino et al., 2013). Further, Rapid Annotation using Subsystem Technology (RAST) version 2.0 (Aziz et al., 2008) was applied for genes of interest. The annotated genes were inspected for probiotic potential. The *de novo* assembled complete genomes were compared using eggnog (evolutionary genealogy of genes: Non-supervised Orthologous Groups)-mapper (Huerta-Cepas et al., 2017) and the associated default settings and Average Nucleotide Identity (ANI) values were obtained using the ANI Calculator tool on EzBiocloud^2^ (Yoon et al., 2017).

### Statistical Analyses

GraphPad Prism 5.0 was used to process data. To compare column to column, unpaired, a two-tailed student’s *t*-test was used. One-way analysis of variance (ANOVA) with Tukey’s post-test was used for multiple comparison, and two way ANOVA was used with Bonferroni post-test for group analysis.

## Results

### Characterization of *L. plantarum* Strains

#### Carbohydrate Fermentation Abilities of *L. plantarum* Strains

Fermentation patterns of carbohydrate sources by each *L. plantarum* strain are noted in Supplementary Table S1. Notable differences in fermentation capability were observed: K6 weakly fermented L-arabinose resulting in a green to blue colorization transition of the API indicator medium instead of yellow, while K2 and K8 were completely negative; K6 fermented raffinose and melizetose while K8 only fermented melizetose, while K2 could not ferment either of the carbohydrates; and turanose was fermented by K6 and K8. Based on the pattern identification through the APIWEB database of BioMérieux, identities (%) of each strain were as follows: K2, 52.0% to *L. plantarum* group 1; K6, 99.4% to *L. plantarum* group 1; K8, 99.5% to *L. plantarum* group 1.

#### Heat Resistance of *L. plantarum* Strains

**FIGURE 1:**
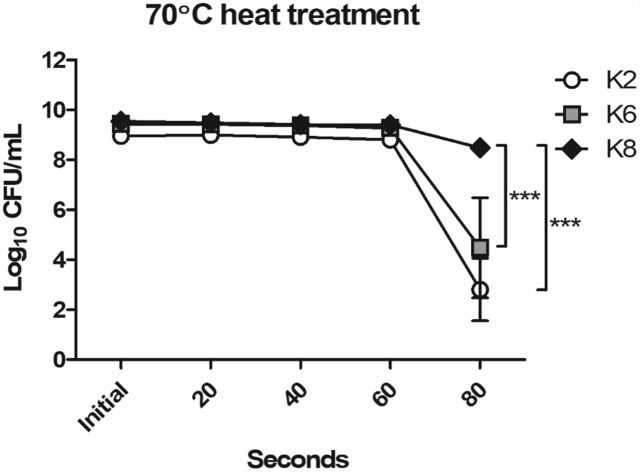
Heat resistance of *Lactobacillus plantarum* strains ATG-K2, ATG-K6, and ATG-K8. CFU counts of triplicate experiments are expressed as mean ± SEM. Statistical significance between compared groups are indicated as ^∗∗∗^*p*< 0.001.

The most resistant strain to 70°C heat treatment for 80 s was K8, followed by K6 and then K2 (Figure 1). As shown in Figure 1, K2 and K6 were unstable and showed low reproducibility in replicate experiments as the error bars indicate. Except for the 80 s time point, all strains showed stable heat resistances up to 60 s. Notably, maximum viable CFU counts of *L. plantarum* strains when cultured overnight in MRS broth were as follows: K2, 9.15 × 10^8^ CFU/mL; K6, 2.83 × 10^9^ CFU/mL; and K8, 3.57 × 10^9^ CFU/mL.

#### Functional Characteristics of *L. plantarum* Strains

The three *L. plantarum* strains showed antibacterial effects against all representative opportunistic pathogens in the disc diffusion assay (Table 1). K2 had the largest diameters of inhibition zones except for tests against EC and PA. Tests were conducted with live bacteria suspensions or CFCS, but only live bacteria suspensions showed inhibition activities.

**Table 1:** Antibacterial effects of *Lactobacillus plantarum* strains ATG-K2, ATG-K6, and ATG-K8 against *Staphylococcus aureus* (SA), *Listeria monocytogenes* (LM), *Streptococcus mutans* (SM), *Streptococcus salivarius* (SS), *Escherichia coli* (EC), *Pseudomonas aeruginosa* (PA), and *Cronobacter sakazakii* (CS) determined by disc diffusion assay.

Strains	SA	LM	SM	SS	EC	PA	CS
*L. plantarum* ATG-K2	5.00 ± 0.18	5.50 ± 0.18	4.75 ± 0.28	4.13 ± 0.11	3.38 ± 0.21	3.75 ± 0.13	6.50 ± 0.18
*L. plantarum* ATG-K6	4.88 ± 0.21	4.75 ± 0.13	4.13 ± 0.11	3.13 ± 0.11	3.50 ± 0.18	4.00 ± 0.18	6.13 ± 0.11
*L. plantarum* ATG-K8	4.25 ± 0.13	5.18 ± 0.21	3.88 ± 0.11	4.00 ± 0.18	3.50 ± 0.18	3.88 ± 0.11	5.88 ± 0.11

*Diameters of inhibition zones are expressed as mean ± SEM in millimeters from four repeated experiments.*

**FIGURE 2:**
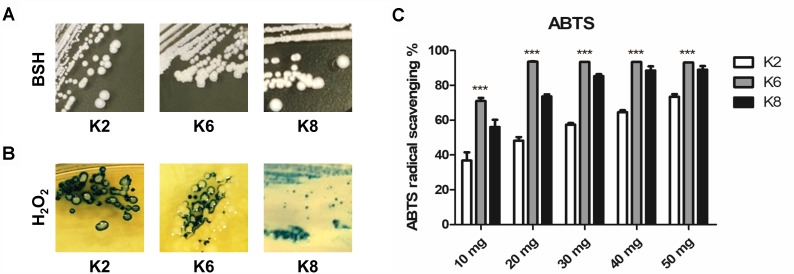
Functional properties of *L. plantarum* ATG-K2, ATG-K6, and ATG-K8 showing **(A)** bile salt hydrolase activity, **(B)** hydrogen peroxide production, and **(C)** ABTS radical scavenging activities of lysates of each strain. Statistical significance ^∗∗∗^ of **(C)** indicates *p*< 0.001 when K6 compared to K2 and K8.

All three strains of *L. plantarum* showed positive BSH activity which was identified by the formation of convex, shell-like, hardened colonies (Figure 2A). H_2_O_2_ production was detected in all three strains of *L. plantarum* based on the blue pigment of O^−^ from TMB reactions in formed colonies and surroundings (Figure 2B). All lysates from the *L. plantarum* strains showed radical scavenging activity and K6 had the highest radical scavenging rate, which reached a plateau at 20 mg/mL concentration of the bacterial lysate, followed by K8 and then K2 (Figure 2C).

### Safety of *L. plantarum* Strains

**FIGURE 3:**
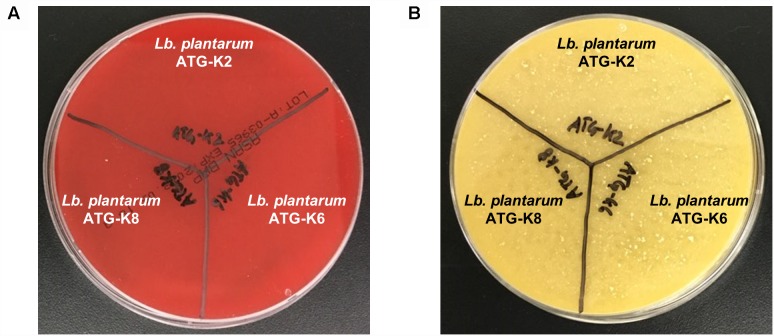
Safety assessment results of *L. plantarum* ATG-K2, ATG-K6, and ATG-K8 of **(A)** hemolysis activity **(B)** biogenic amine production.

There was no hemolysis activity detected for all three strains of *L. plantarum* as is indicated by a lack of clear zone formation (Figure 3A). Also, there was no color change of the test medium in the biogenic amine production test which implies that no histidine, tyramine, putrescine, or cadaverine was produced by any of the three strains of *L. plantarum* (Figure 3B). Putrescine derived from agmatine was not able to detect due to the composition of the test medium suggested by Bover-Cid and Holzapfel (1999).

All three strains met the requirements of MIC cut-off values suggested by the EFSA guideline on antibiotic susceptibility of lactic acid bacteria (Table 2). Although the EFSA guideline does not require vancomycin and streptomycin susceptibilities, a 94–194 μg/mL MIC for vancomycin and streptomycin was observed from antibiotic susceptibility tests of three strains (data not shown in Table 2).

**Table 2:** Antibiotic susceptibility of *L. plantarum* strains ATG-K2, ATG-K6, and ATG-K8.

Strains	AMP	GEN	KAN	CD	ERY	TET	CM
*L. plantarum* ATG-K2	0.094	12	12	1	0.25	6	4
*L. plantarum* ATG-K6	0.094	12	16	0.75	0.125	3	2
*L. plantarum* ATG-K8	0.125	16	16	1	0.19	4	0.75

*Results are expressed as minimum inhibitory concentrations (MIC, μg/mL) determined by E-test strips. Abbreviation of antibiotics are as follows: AMP, ampicillin; GEN, gentamicin; KAN, kanamycin; CD, clindamycin; ERY, erythromycin; TET, tetracycline; CM, Chloramphenicol.*

### Antimicrobial Properties of *L. plantarum* Strains Against CA and GV

#### Inhibitory Effects of *L. plantarum* Strains Against Pathogens

**FIGURE 4:**
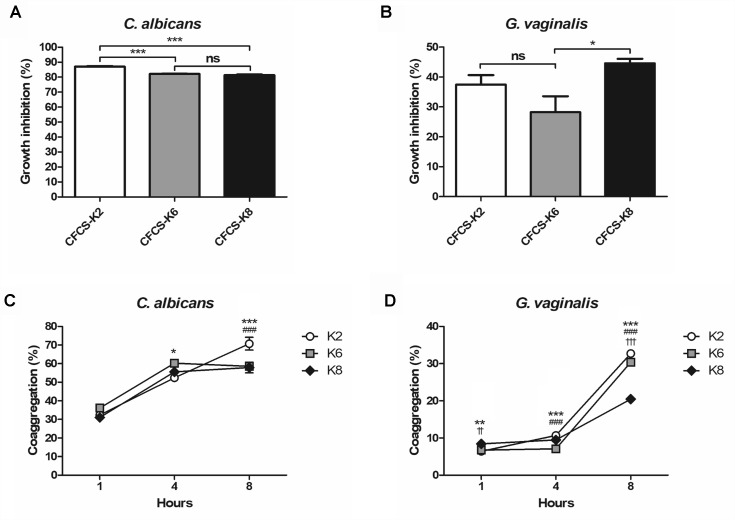
Growth inhibition rates against **(A)** *C. albicans* and **(B)** *G. vaginalis* by cell free culture supernatant (CFCS) of *L. plantarum* ATG-K2, ATG-K6, and ATG-K8. Coaggregation of each strain of *L. plantarum* ATG-K2, ATG-K6, and ATG-K8 reacted with **(C)** *C. albicans* and **(D)** *G. vaginalis*. Data are expressed as mean ± SEM. Statistical significance in the figures are shown as follows: K2 vs. K6, ^∗^*p* < 0.05, ^∗∗^*p* < 0.01, ^∗∗∗^*p* < 0.001; K2 vs. K8, ^###^*p* < 0.001; K6 vs. K8, ^††^*p* < 0.01, ^†††^*p* < 0.001.

CFCS of each *L. plantarum* strain showed growth inhibition toward CA and GV (Figure 4A,B). Against CA at the 24 h mark, K2, K6, and K8 showed 87.05 ± 0.38%, 82.11 ± 0.25%, and 81.34 ± 0.42%, respectively (Figure 4A). After 48 h of incubation, GV growth had inhibition rates by CFCS of K2, K6, and K8 of 37.49 ± 3.17%, 28.25 ± 5.33%, and 44.60 ± 1.51%, respectively (Figure 4B). The coaggregation rate of K2, K6, and K8 were 70.73 ± 3.41%, 58.57 ± 1.32%, and 57.88 ± 2.86%, respectively, when reacted with CA after 8 h (Figure 4C). In comparison to coaggregation with CA, coaggregation rates between GV and each *L. plantarum* strain were lower than that in the corresponding CA experiment. The coaggregation rates of K2, K6, and K8 were 32.72 ± 0.19%, 30.39 ± 0.13%, 20.45 ± 0.42%, respectively, when reacted with GV after 8 h (Figure 4D).

#### Killing Effects of *L. plantarum* Strains Against Pathogens

**FIGURE 5:**
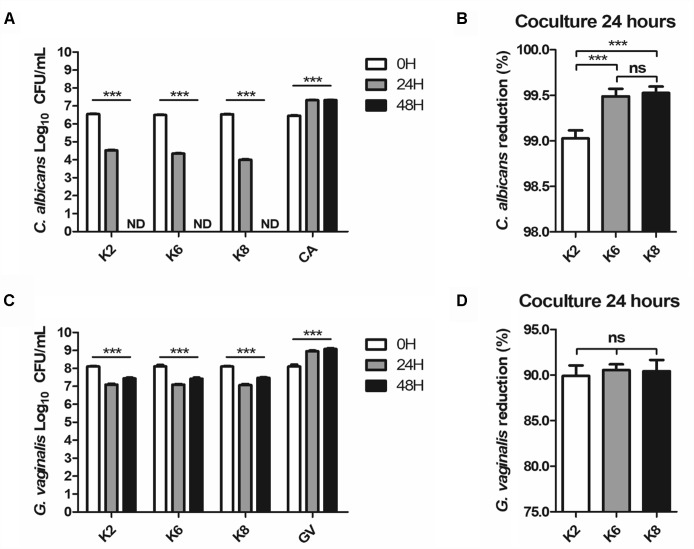
**(A,B)** *C. albicans* and **(C,D)** *G. vaginalis* killing effects of live *L. plantarum* ATG-K2, ATG-K6, and ATG-K8 determined by a coculture method. Log_10_ CFU/mL of each target pathogen measured in 24 h intervals are shown in **(A,C)**. Reduction rates of each target pathogen at the 24 h mark are shown in **(B,C)**. Data are expressed as mean ± SEM for four replicated experiments. Statistical significances are shown as ^∗^*p* < 0.05, ^∗∗^*p* < 0.01, and ^∗∗∗^*p* < 0.001.

Complete disappearance of CA after 48 h of coculture was observed for all strains (Figure 5A). At the 24 h mark, the CA reduction rate by K2, K6, and K8 were 99.03 ± 0.06%, 99.49 ± 0.04%, and 99.53 ± 0.02%, respectively (Figure 5B). K6 and K8 showed statistically significant CA reduction rates compared to K2. GV was reduced by K2, K6, and K8, at rates of 87.37 ± 1.03%, 92.50 ± 0.54%, and 93.71 ± 1.10%, respectively, and no significant differences between each test group were found (Figure 5D). However, GV growth was detected at the 48 h mark in a roughly doubled CFU/mL after being reduced to 90% on average by each *L. plantarum* strain (Figure 5C).

### Immunomodulatory Effects of *L. plantarum* Strains in a Murine Macrophage Model

#### Induction of Innate Immunity-Related Cytokines by Lysates of *L. plantarum* Strains

**FIGURE 6:**
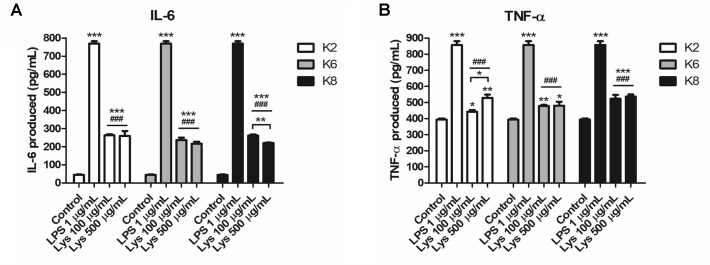
Induction of **(A)** IL-6 and **(B)** TNF-α by lysates of *L. plantarum* ATG-K2, ATG-K6, and ATG-K8 in RAW264.7 murine macrophage cells. The means of triplicated wells are shown from duplicated experiments ± SEM. Statistical significance in the figures are as follows: compared to control, ^∗^*p*< 0.05, ^∗∗^*p*< 0.01, and ^∗∗∗^*p*< 0.001; compared to the LPS group ^###^*p*< 0.001. Significant differences between doses in the same group are shown with directional lines as ^∗^*p*< 0.05 and ^∗∗^*p*< 0.01.

RAW264.7 cells produced significantly increased IL-6 and TNF-α upon treatment with each *L. plantarum* strain lysate compared to the control and was significantly lower than that of LPS-treated group (Figure 6). Each cytokine concentration was increased to both 100 and 500 μg/mL lysate concentrations. However, there was no significant difference between induced IL-6 concentrations by the same dose of lysates (Figure 6A). For 100 μg/mL lysate treatment, the K8 lysate-treated group (523.47 ± 23.78 pg/mL of TNF-α, *p*< 0.05) showed significantly higher concentrations of TNF-α compared to that of the K2 lysate (443.25 ± 10.50 pg/mL of TNF-α) and K6 lysate (477.81 ± 7.57 pg/mL of TNF-α)-treated groups (Figure 6B). In the 500 μg/mL lysate dose groups, there were no significant differences between any *L. plantarum* lysate treated groups for both cytokines.

#### Enhanced IL-10 Production by Lysates of *L. plantarum* Strains Against LPS Challenge

**FIGURE 7:**
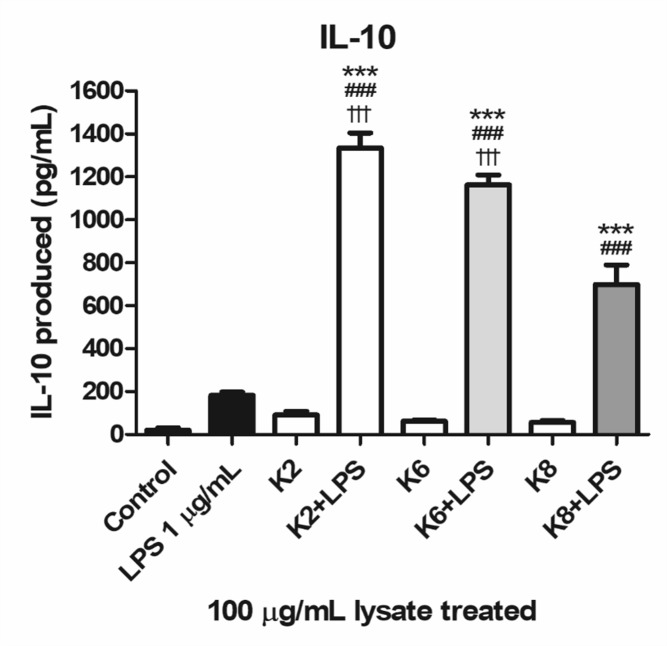
Induction of IL-10 lysates of *L. plantarum* ATG-K2, ATG-K6, and ATG-K8 in RAW264.7 murine macrophage cells upon LPS challenge. LPS (1 μg/mL) challenged groups are marked as +LPS in X axis labels. The means of triplicated wells are shown as a representative of duplicated experiments ± SEM. Statistical significance in the figures are as follows: compared to control, ^∗^*p*< 0.05, ^∗∗^*p*< 0.01, and ^∗∗∗^*p*< 0.001; to the LPS group, ^##^*p*< 0.01, ^###^*p*< 0.001; to the K8+LPS group ^†††^*p*< 0.001.

All three lysates of each *L. plantarum* strain were induced with a significantly higher concentration of IL-10 when treated with LPS compared to the control and LPS-only treated group (Figure 7). Significantly increased IL-10 production was observed in K2 and K6 lysates treated with 1 μg/mL LPS compared to K8 lysate treated with LPS. There was no statistically significant difference between K2 and K6 lysates treated with LPS.

### Whole Genome Analyses

#### Whole Genome Assembly and Annotation

**FIGURE 8:**
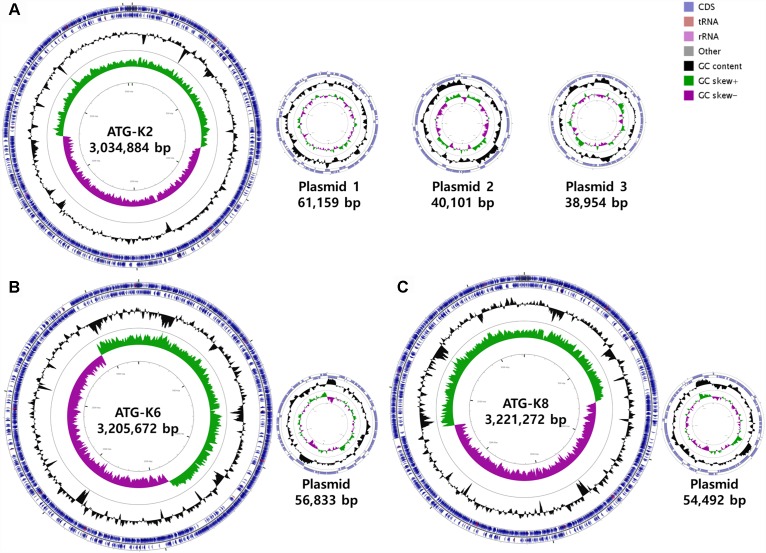
Circular maps of **(A)** *Lactobacillus plantarum* ATG-K2, **(B)** *L. plantarum* ATG-K6, and **(C)** *L. plantarum* ATG-K8 complete genomes.

The genomes of *L. plantarum* strains were sequenced with the PacBio SMRT platform with resulting 330× coverage for K2, 384× for K6, and 432× for K8. The genome of K2 was assembled into one circular chromosome and three plasmids, while the genomes of K6 and K8 were assembled into one circular chromosome and one plasmid (Figure 8). The total genome size of K8 (3,275,764 bp) is slightly larger than those of K2 (3,175,098 bp) and K6 (3,262,505 bp). The protein coding sequences were predicted using NCBI PGAAP and deposited in GenBank with accession numbers GCA_003597635.1 (K2), GCA_003597595.1 (K6), and GCA_003597615.1 (K8). A total of 3,114 open reading frames (ORFs) were predicted from the genome of the strain K2; 2,857 are protein coding genes and 85 are RNA genes. A total of 3,178 ORFs were predicted from the genome of the strain K6; 2,999 are protein coding genes and 87 are RNA genes. Finally, a total of 3,186 ORFs were predicted from the genome of the strain K6; 2,999 are protein coding genes and 87 are RNA genes (Table 3).

**Table 3:** General features of *L. plantarum* strains ATG-K2, ATG-K6, and ATG-K8.

Attribute	Value of strain ATG-K2	Value of strain ATG-K6	Value of strain ATG-K8
Total genome size (bp)	3,175,098	3,262,505	3,275,764
Chromosome size	3,034,884	3,205,672	3,221,272
Plasmid number	3	1	1
DNA G+C (%)	45.19	44.54	44.54
Total genes	3,114	3,178	3,186
Protein coding genes	2,857	2,999	2,999
rRNA genes	16	16	16
tRNA genes	65	67	67
ncRNA genes	4	4	4
Pseudo genes	172	92	100
GenBank accession	GCA_003597635.1	GCA_003597595.1	GCA_003597615.1

#### Comparative Genomics and Probiotic Component Analysis

**FIGURE 9:**
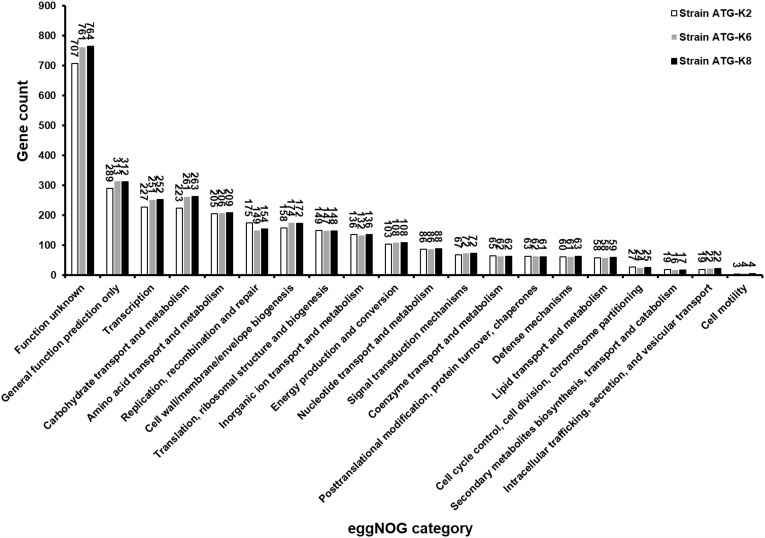
Numbers of proteins encoded by *Lactobacillus plantarum* ATG-K2, *L. plantarum* ATG-K6, and *L. plantarum* ATG-K8 annotated with the corresponding eggNOG functional category.

Based on ANI analyses, K2 showed 96.30 and 96.12% sequence identity with K6 and K8, respectively. Strain K6 and K8 showed 99.92% sequence identity. All three strains were predicted to be non-human pathogens with PathogenFinder. Coding sequences were identified and grouped in COG classes (Figure 9). The coding sequences were categorized as by their involvement in carbohydrate transport and metabolism (7.9–8.8%); transcription (8.0–8.5%); amino acid transport and metabolism (6.9–7.2%); translation, ribosomal structure and biogenesis (4.9–5.2%); cell wall, membrane, envelope biogenesis (5.6–5.9%); and replication, recombination and repair of nucleic acids (5.0–6.2%). K2 (counted number: 223) showed a lower gene number for carbohydrate transport and metabolism compared with K6 (counted number: 261) and K8 (counted number: 263), while K2 (counted number: 175) showed a higher gene number for replication, recombination and repair compared with K6 (counted number: 149) and K8 (counted number: 154).

## Discussion

Three strains of *L. plantarum* in the present study, namely K2, K6, and K8, demonstrated BSH activity, H_2_O_2_ production, immunomodulatory effects, and antimicrobial functions especially against CA which revealed potential probiotic effects of these strains. Also, safety assessment results support that K2, K6, and K8 are safe for practical use with along with the safety of the *L. plantarum* species in general described by De Vries et al. (2006).

From a microbial perspective, BSH activity is important for secondary bile salt metabolism which may contribute to bile resistance of bacteria and benefit cholesterol metabolism of the host by lowering the host’s bile salt concentration, but excessive deconjugated bile salt may interfere digestive activity or intestinal health of the host, deconjugated bile salt production of BSH positive strains should be further investigated in quantitative manner (Begley et al., 2006). H_2_O_2_ produced by *L. plantarum* strains of the present study may be involved in killing opportunistic pathogens (De Vuyst and Vandamme, 1994). In addition to H_2_O_2_ production, radical scavenging activity from produced H_2_O_2_ might be an important factor for their own survival (Condon, 1987) as well as removing excessive radicals produced in local sites of the host through their oxidative enzymes (Lin and Yen, 1999). Among antimicrobial activities against several pathogens, anti-CA effects can benefit prevention of candidiasis in mucosal sites which is the first line of defense in the host. Particularly, coaggregation ability of *L. plantarum* strains with CA can be synergic with growth inhibition, H_2_O_2_, and killing effects depending on proximity. Although relatively weaker antimicrobial activities against GV were observed compared to anti-CA effects, it may still contribute to a prevention of BV taken together with antibacterial effects toward other opportunistic pathogens. Also, anti-CA effects also may benefit vaginal health since CA is also a pathogen that causes vaginitis (Sobel et al., 1998). In short, microbial properties of *L. plantarum* strains in the present study suggest the potential of the strains as probiotics for mucosal health.

From an immunology perspective, induction of IL-6 and TNF-α in macrophages by lysates of K2, K6, and K8 implicates that innate immunity is enhanced. IL-6 is a cytokine which is involved in activation of macrophages, recruitment and chemotaxis of neutrophils, activation of inflammatory responses and regulation of the immune response (Fielding et al., 2008; Scheller et al., 2011). TNF-α is another important cytokines that has roles in anti-tumor and proinflammatory responses, pyrexia, inhibition of viral replication, and activation of monocytes (Vujanovic, 2011). However, these proinflammatory cytokines must be tightly regulated to not result in pathological excessive inflammation. For regulation of such excessive inflammatory responses, IL-10 needs to be produced. IL-10 is an anti-inflammatory and regulatory cytokine that is produced by dendritic cells, macrophages, and regulatory T cells, and thus elevated concentration of IL-10 will suppress excessive activation of proinflammatory responses (Fiorentino et al., 1991; Saraiva and O’Garra, 2010). As shown in the co-treatment of LPS and lysate of *L. plantarum* strains in the present study, IL-10 production is enhanced when macrophages are activated by LPS. In addition, the presence of IL-6 and TNF-α is important for enhanced production of IL-10 which as demonstrated in *in vitro* human cell experiments (Daftarian et al., 1996). Interestingly, enhanced IL-10 production was lower in the K8 lysate-treated group compared to that of the K2 and K6 lysate treated groups, which it may be related to the formation of differential immune states or “immune tones” at a strain-specific level (Beck et al., 2016). However, this needs to be examined in a more complex animal model system to dissect strain-specific immunomodulatory effects in further studies. As macrophages have essential roles in mucosal immunity (Mowat and Bain, 2011; Ginhoux and Jung, 2014), the results of macrophage experiments supports that the enhanced host immunity and probiotic effects of *L. plantarum* strains in the present study might be synergistic.

From a genomic perspective, although all three strains were of the same species, K2 showed smaller genome size and different compositions compared to the other two strains, which implies the importance of genomic studies to distinguish and explore characteristics of microorganisms at a strain level. For example, lower numbers of carbohydrate transport and metabolism, and transcription-related genes in the K2 strain compared to other strains may influence maximum growth (CFU/mL), which was approximately 30% of that of K6 and K8 as noted in the results of heat resistance examination. An absence of potential virulence factors reconfirms the safety of *L. plantarum* strains. On the other hand, antimicrobial activities of three strains were positive but to different degrees which may relate to differential composition of the plantaricin biosynthesis gene cluster (Li et al., 2016). Varying degrees of immunological responses of RAW264.7 cells to the each strain may be due to differential activation of pattern recognition receptors due to differences in cell membrane components, or production of different metabolites (Hu et al., 2011; Xue et al., 2014). In short, genomic analyses of three strains strongly support the uniqueness of each strain. These factors should be considered in future genome-wide studies of these three strains.

## Conclusion

In conclusion, *L. plantarum* strains ATG-K2, ATG-K6, and ATG-K8 in the present study demonstrated their potentials for mucosal health improvement in both microbiological and immunological contexts which may result in synergistic effects by influencing both microbial and host factors.

## Author Contributions

BB contributed to the experimental designs, experimented on the microbiological assays and the *in vitro* assays with cells, the writing and editing of the manuscript, and agreed to be listed as a corresponding author. G-SP contributed to the genome sequencing and analyses, and the writing of the manuscript. YL experimented on the *in vitro* assays with cells. SI oversaw the culture and management of *L. plantarum* strains. DJ experimented on the microbiological assays. JK contributed to the experimental designs, the editing of the manuscript editing, and agreed to be listed as a corresponding author.

## Conflict of Interest Statement

All authors were employed by AtoGen Co., Ltd.

## Footnotes

1 https://github.com/PacificBiosciences/SMRT-Analysis

2 http://www.ezbiocloud.net/
