# Supplementary material for: Whole Genome Analysis of Lactobacillus plantarum Strains Isolated From Kimchi and Determination of Probiotic Properties to Treat Mucosal Infections by Candida albicans and Gardnerella vaginalis
Source: Front Microbiol. 2019 Mar 6;10:433. doi: 10.3389/fmicb.2019.00433 (PMC6414439; doi:10.3389/fmicb.2019.00433)
Supplement: Supplementary file 1 [file Table_1.DOCX]

**Supplementary materials**

Table S1. Carbohydrate fermentation patterns of *Lactobacillus plantarum* strains ATG-K2, ATG-K6, and ATG-K8. Fermentation results are indicated as follows: +, positive; w, weak positive; -, negative.

| Carbohydrates | *L. plantarum* ATG-K2 | *L. plantarum* ATG-K6 | *L. plantarum* ATG-K8 |
| --- | --- | --- | --- |
| Glycerol | - | - | - |
| Erythritol | - | - | - |
| D-Arabinose | - | - | - |
| L-Arabinose | - | w | - |
| Ribose | + | + | + |
| D-Xylose | - | - | - |
| L-Xylose | - | - | - |
| Adonitol | - | - | - |
| Methyl-βD-Xylopyranoside | - | - | - |
| Galactose | + | + | + |
| Glucose | + | + | + |
| Fructose | + | + | + |
| Mannose | + | + | + |
| Sorbose | - | - | - |
| Rhamnose | - | - | - |
| Dulcitol | - | - | - |
| Inositol | - | - | - |
| Mannitol | + | + | + |
| Sorbitol | + | + | + |
| Methyl-αD-Mannopyranoside | - | - | - |
| Methyl-αD-Glucopyranoside | - | - | - |
| N-Acetylglucosamine | + | + | + |
| Amygdalin | + | + | + |
| Arbutin | + | + | + |
| Esculin | + | + | + |
| Salicin | + | + | + |
| Cellobiose | + | + | + |
| Maltose | + | + | + |
| Lactose | + | + | + |
| Melibiose | + | + | + |
| Sucrose | + | + | + |
| Trehalose | + | + | + |
| Inulin | - | - | - |
| Melezitose | - | + | + |
| Raffinose | - | + | - |
| Starch | - | - | - |
| Glycogen | - | - | - |
| Xylitol | - | - | - |
| Gentiobiose | w | w | w |
| Turanose | - | + | + |
| Lyxose | - | - | - |
| Tagatose | - | - | - |
| D-Fucose | - | - | - |
| L-Fucose | - | - | - |
| D-Arabitol | - | - | - |
| L-Arabitol | - | - | - |
| Gluconate | w | w | w |
| 2-keto-gluconate | - | - | - |
| 5-keto-gluconate | - | - | - |
